# HAPPINESS IN CONVENTIONAL VERSUS RETIREMENT HOMES: IMPORTANCE OF THRIVING AND COMMUNITY INTEGRATION

**DOI:** 10.1093/geroni/igae098.1446

**Published:** 2024-12-31

**Authors:** Melanie Levasseur, Daniel Naud, Martine Lagacé, Marie-Ève Bédard, Shannon Hebblethwaite

**Affiliations:** Université de Sherbrooke, Sherbrooke, Quebec, Canada; Université de Sherbrooke, Sherbrooke, Quebec, Canada; University of Ottawa, Ottawa, Ontario, Canada; Cégep de Drummondville, Drummondville, Quebec, Canada; Concordia University, Montreal, Quebec, Canada

## Abstract

Since aging sometimes implies a change in living environment, it is important to better understand happiness and associated factors such as thriving, social participation, community integration and ageism according to residential settings. This study aimed to compare the happiness of older women and men living in a conventional dwelling (CD) and in an independent living facility (ILF), and examined its associations with thriving, social participation, community integration and ageism (self-directed and discrimination). A cross-sectional survey was carried out on a random sample of 509 adults aged 75 and older living in DC and 470 in ILF. Regressions stratified by residential setting and gender examined associations between happiness, thriving and social participation, testing for the moderating effect of ageism and community integration. Participants were 81.3+/-5.3 on average, and two-thirds were women. Controlling for age, living situation and health, the happiness of older adults living in CD and ILF was similar (69.7+/-14.0 vs 68.8+/-18.4; p=0.262). Superior thriving was strongly associated with greater happiness, regardless of residential setting or gender. Higher social participation was associated with greater happiness among women in ILF. For both settings, self-directed ageism was associated with lesser happiness, but discrimination was associated with lesser happiness only in women living in ILF. Higher community integration was associated with greater happiness in both settings, except for men in ILF. According to these results, future studies should evaluate interventions aimed at facilitating thriving and reducing ageism may improve happiness of older adults.

